# Review of Current Neurological Matters

**Published:** 1877-09-20

**Authors:** C. C. Vanderbeck


### SOUTHERN MEDICAL RECORD.

VOL. VII. ATLANTA, GA., SEPTEMBER 20, 1877. No. 9.

THOMAS S. POWELL, M.D.,

W. T. GOLDSMITH, M. D.,

R. C. WORD, M. D.,

} EDITORS.

R. C. WORD, Business Manager.

SUBSCRIPTION: TWO DOLLARS PER ANNUM, IN ADVANCE.

☞ All communications, and letters on business connected with THE RECORD for 1877 must be addressed to the Business Manager.

### Original and Selected Articles.

### REVIEW OF CURRENT NEUROLOGICAL MATTERS.

CONDENSED BY C. C. VANDERBECK, M. D.

(Concluded.)

CONVULSIONS OF CHILDREN. — Dr. Trezevant, of Columbia, has used physostigma in several cases of infantile convulsions, with excellent results. He was led to use it from the suggestion given by the great benefit obtained from the drug in a case of tetanus. The spinal system being in excess over the cerebral system in children, we possess in this medicine "an agent capable of neutralizing and keeping in check this predominance of the spinal over the cerebral system." The dose he gave was one-thirty-second of a grain of the alcoholic extract for a child five months old, and repeated in two hours if necessary. Two doses were given. To a boy, aged 11 months, he gave three minims of a solution of this kind: One grain of the extract of the bean, rubbed up with thirty minims each of glycerine and water. Dr. McLaurin, of Edinburg, cured a "remarkable case of tonic convulsions, which persisted for many months, the fits recurring several times a day." Every remedy was tried in vain, and the patient was getting continually worse. He now used physostigma. The dose was gradually increased, until four grains of the bean was taken four times a day. "It reduced the pulse to 58, and excited gastric uneasiness." The pupil was unaffected. The intervals between the paroxysm became longer, and at the end of six weeks ended altogether. The same gentleman reports a case, in the London *Lancet*, of a little girl four and a half years old, who had convulsions several times a day for nine months. Not a single attack occurred after the first dose of the medicine.—*Richmond Med. Monthly*.

Why should physostigma not be more extensively tried for various convulsive diseases? Might not a trial of it be made in puerperal eclampsia, and in epilepsy?

